# Editorial: new Editor-in-Chief and the 360th anniversary of *Philosophical Transactions*

**DOI:** 10.1098/rsta.2024.0557

**Published:** 2025-01-16

**Authors:** C. Richard A. Catlow

**Affiliations:** ^1^ Department of Chemistry, University College London, 20 Gordon Street, London WC1HOAJ , UK; ^2^ School of Chemistry, Cardiff University, Park Place, Cardiff CF10 3AT, UK

**Keywords:** maths, engineering, physics, chemistry, computer science, Earth science

­

It is a pleasure to be writing my first editorial as Editor-in-Chief of the journal, taking over from Professor John Dainton, whose excellent stewardship has contributed greatly to the journal’s recent success. My own scientific fields of computational, materials and catalytic chemistry have featured strongly in the journal, and I am proud to have published in it regularly since my first *Philosophical Transactions A* paper in 1991 on the chemical nature of fission products in uranium dioxide nuclear fuels [1]. I have also been a Guest Editor of several of the journal’s theme issues, most recently on ‘Green Carbon for the Chemical Industry of the Future’ [2]. Guest editing a *Philosophical Transactions A* theme issue is always a rewarding experience.

It is an auspicious year to be starting my term as Editor-in-Chief, as in 2025, we are celebrating the 360th anniversary of the first issue of *Philosophical Transactions of the Royal Society*—a landmark in the history of science and of scientific publishing [3]. In its extraordinarily rich and diverse history, the journal has communicated some of the most outstanding scientific discoveries. Examples from the early years in what we now call the ‘physical sciences’ include Isaac Newton’s theory of light and colours in 1671 [4], and Thomas Bayes’ ‘doctrine of chances’ in 1760 [5]. Our journal was also the first to publish the astronomical discoveries of Caroline Herschel in 1787 [6], the first time the scientific work of a woman was fully credited in any scientific publication throughout the world (stay tuned throughout 2025 as the Royal Society will also be celebrating the 80th anniversary of the first female scientists to be elected to the Fellowship). Later periods saw the publication of James Clerk Maxwell’s full ‘dynamical theory of the electromagnetic field’ in 1865 [7].

After the *Philosophical Transactions* was divided into two strands in 1887, the *Philosophical Transactions A* received an increasing number of submissions of articles in the physical sciences, including Dyson, Eddington and Davidson’s ‘Determination of the deflection of light by the sun’s gravitational field, from observations made at the total eclipse of 29 May 1919’ and Dame Kathleen Lonsdale’s ‘Divergent-Beam X-Ray Photography of Crystals’ published in 1947 [8,9].

The journal has indeed played a key role in the development of scientific knowledge as stated by the President of the Royal Society in 1832: *‘[The] Transactions contain records of almost every important discovery in natural philosophy; of almost every experimental inquiry which has been most remarkable for its difficulty, delicacy, or importance; and of almost every original speculation which has most contributed to the advancement of science.’* [10]

The distinguished past of *Philosophical Transactions* is matched by its dynamic presence at the forefront of current science, as illustrated by several recent issues of the journal: ‘Delivering Fusion Energy – The Spherical Tokamak for Energy Production (STEP)’[11]; ‘The effectiveness of non-pharmaceutical interventions on the COVID-19 pandemic: the evidence’[12]; and ‘Cognitive artificial intelligence’ [13].

To what does the journal owe its longevity and success? First, to its core values of scientific integrity; of which, peer review is a key promoter and protector. As is well known, peer review is under great pressure, owing to a number of factors including reviewer fatigue and growing general pressures on the scientific community. Interesting new approaches are being explored, including ‘open peer review’, but it will remain a key component of high-quality scientific publishing. As I repeatedly say to (especially younger) colleagues, we should view peer review not as an adversarial process but as a means for improving and protecting the quality of our published work and the scientific record.

A second key feature of the journal has been its flexibility and adaptability. While retaining its core values, it has changed as science and scientific communication has changed. It is flexible in the type of articles it publishes, accepting original research and reviews, or blends of the two. We welcome proposals for issues from all members of the scientific community, and indeed we would be very pleased to receive more proposals for journal issues from early-career scientists. We also hope that the proposals we receive will reflect the very broad scope of the journal which encompasses all areas of physical science, engineering and mathematics. To find out more about submitting a proposal to the journal, visit https://royalsocietypublishing.org/rsta/submit-proposal.

Scientific publishing is under both pressure and scrutiny. Journal proliferation and the growth of predatory publishing practices are clear threats to scientific integrity, as is the crude and uncritical use of publication metrics as proxies for quality. Royal Society Publishing in general, and *Philosophical Transactions* in particular, will continue to stand for integrity, inclusiveness and rigour in scientific publishing [14].
